# 

**Published:** 1857-10

**Authors:** 


*Coiled Gold for Filling Teeth.*—We have received an interesting artiticle from Dr. C. T. Cushman, of Columbus, Ga., on the Use of Coiled Gold for Filling Teeth. It came to hand, however, too late for publication in the present number. It shall appear in our next issue.

*St. Louis Society of Dental Surgeons.*—We omitted to acknowledge our indebtedness in our last number to the Editors of the Dental Register of the West, for a proof sheet of the report of the proceedings sent to us in advance of the publication of their Journal. We appended such acknowledgment to the report, but it was afterwards separated from the report with the intention of placing it under the editorial head, but was unintentionally left out. We think it due to the courtesy of Drs. Taft and Watt, to make this statement, as they incurred the trouble of procuring the report.

*Omitted.*—We are compelled to omit several notices designed for the present number for want of time to prepare them, it being issued in advance of the regular time for its appearance.

### INDEX.—VOL. VII.

PAGE.

A.

Ammonia in Blood, . . . 143
Anæsthetics, . 144, 254, 261
Austin, Dr., on Cheoplasty, 145
Arctic Explorations: short notice of Dr. Kane's, . . 151
Aluminium, . . . . 193
Artificial Piece made to Replace Inferior Maxillary Bone, 199
Absorption of Roots of Permanent Teeth, . . . . . 221
Alcohol and Tobacco, Physiological Effects of, . . . 229
Antrum Pylori, . . . 292
American Dental Convention, . 300, 451, 465 595
Affections Determined by the Wisdom Teeth, . . 339
American Medical Association, 419
Alveolar Abscess, . . . 448
Amylene, . . . . 452
Adhesive Gold Foil, a treatise on the use of, . . . . 598
Arthur, Robert, on the Use of Adhesive Gold Foil, . 598

B.

Baths, on the Use and Abuse of Chemical . . . . 98
Blundell, Painless Tooth Extraction, . . . 151
Blood, Lecture on the Circulation of the, and the Theories of Generation. By Christopher Johnston, . . 157
Blackman George C., Removal of the Entire Lower Jaw for Osteo-Sarcoma, . . 249
Blindness, Color, . . . 297
Brown, A. J., Cheoplasty and its Results, . . . 555

PAGE.

C.

Climate, Influence of on the Human Teeth, . . . 74
Chemical Baths, Use and Abuse of, . . . . . 98
Cerebellum, Function of the, 143
Chemistry of Respiration, 143
Col. of Dent. in England, 152, 389
Chloroform, Poisoning by taking internally, . . . 264
Chemistry of the Mouth, . 281
Cheoplastic Method of Mounting Teeth, 302, 450, 550, 555
Coiled Gold Foil for Filling Teeth, . . . . . 600
Cheoplastic Dentistry, . 550
Cheoplasty and its Results, 555

D

Dental Pathology, . . 14
Dental Art, Works on the, 32
Dentition Infantile, . 70
Dentine, Action of Topical Remedies on Inflamed, . . . 112
Dentine, Sensitive, . . . 126
Donations to the Baltimore College of Dental Surgery, 156
Dent Soc., North Carolina, 148, 154
Dana, Geology of the Pacific, &c.150
Development of the Human Teeth, Physiological Reflections on the . . . 310
Demorey, Obs. on Salivary Calculus in the Duct of Wharton, 344
Disarticulation and Removal of the Lateral half of the Lower Jaw, . . . . . 432
Denudation of the Dental Pulp, 78
Dentine and Enamel, on the Development of the . . 562

PAGE.

E.

Exostosis, Diseased Medullary of the Left Upper Max. Bone, 68
Excavators, . . . . 276
Extraction of a Tooth, Death from . . . . . 452

F.

Facial Nerve, Paralysis of the, 79
Forceps, Improved, . . . 106
Filling Labial Surfaces of Upper Incisors, . . . . 322
Forceps, the Adapted, . . . 349
Fluorine in the blood, . 599

G.

Gregory E., Paralysis of the Facial Nerve, . . . 79
Gangrenous Degeneration of the Cheek and Gums, with Necrosis and Exfoliation, &c., 133
Geology of the Pacific, . . . 150
Grinding Apparatus, . 155
Generation, Theories of, &c., 157
Gold Plastic, . . . . 299

H.

Hyoid Bone, Removal of a Tumor from the, . . . 132
Harris, C. A., Letter to Dr. Austen in reference to the Cheoplastic Method, . 145
Heyfelder, Oscar, on the Resection of both Upper Jaw Bones, 206
Hammond, Wm. A., on Alcohol and Tobacco, . . . 229
Hayes' Method of Mounting Artificial Teeth, . . . 269
Hygiene Boarding School, 298
Hullihen, Dr. S. P., Obituary, 303
Hemicrania from a Carious Tooth and Necrosed Root, 449, 453
Histology—its importance, &c., 573

I.

Introductory Lecture to the Course upon Microscopical and Comparative Anatomy, 3
Infantile Dentition, . . . 70
Improvements in Adjustable Punches, . . . . 147

PAGE.

Index to Vol. Sixth, . . . 155
Isaacs, C. E., Removal of Half of the Lower Jaw, . 432

J.

Johnston, Dr. Chris., Introduct. Lecture to the Course upon Microscop. and Compar. Anat. of the Teeth, in the Baltimore College of Dental Surgery, 3
Johnston Dr. Chris., Letter from, on a Microscopical preparation, 66
Johnston, Dr. Chris., Lecture upon the Discovery of the Circulation of the Blood, and the Theories of Generation, 157
Johnston, Dr. Chris., Contribution to the Minute Anatomy of the Teeth, . . . 348
Jaw Bones, on the Resection of both Upper, . . . 206
Jaw, Removal of the entire Lower for Osteo-Sarcoma, . 249
Jaw, Lower, Disarticulation and Removal of nearly the Lateral half of the . . . 432

L.

Lamp, Pratt's Patent Safety, and Feeder, . . . 155
London Quarterly Journal of Dental Science, . . 450
Liver, Minute Anatomy of, 286
Lent, Edward, on the Development of Dentine and Enamel, 562
Levison, J. L. Case of Hemicrania, from a Carious Tooth and Necrosed Fangs, with a highly vascular condition of the pulp cavity, . 449, 453

M.

Medullary Exostosis of the Left Upper Maxillary, . . 68
Micrometer Object-Finder, 84, 92
Maclean, Samuel, Improved Forceps, . . . . . 106
Models and Casts, . . 272
Mississippi Valley Dental Depot, 303

N.

North Carolina Dent. Soc. 148, 154

PAGE.

O.

Organic Union of Contiguous Teeth, . . . . 111
Organization of the St. Louis Dental Society, . . . 203
Odontological Society in London, . . . . 301, 381
Obituaries of Drs. Hullihen and Ure, . . . 303, 304
Osseous Union of Two Supernumerary Teeth, . . . 451
Osseous Union of Teeth, . 600

P.

Paralysis of the Facial Nerve. By E. Gregory, . . . . 79
Punches, Improvement in for Setting Artificial Teeth, . 147
Polishing Stones, . . 154
Paris, Dr. . . . . . 154
Pratt's Patent Safety Lamp and Feeder, . . . 155
Poisoned Sausages, . . 223
Plastic Gold, . . . 299
Palatine Region, Essay on Tumors in. By Dr. Parmentier, 324
Plastic Operations for the Restoration of the Lower Lip. By Thos. Teale, . . 442
Plating Silver Bases, on which Artificial Teeth are Mounted with Gold, . . . . 451
Parotid Region, Tumors of the, 587

R.

Rymer S. Lee. Denudation of the Dental Pulp, . . 78
Report of the Committee appointed by the Microscopical Society, for the purpose of ascertaining the most convenient form for indicating the position of objects under the microscope, 92
Richardson Dr. J., Gangrenous Degeneration of the Cheek and Gums, etc., . . . 133

PAGE.

Retzius, Prof. A., on the Antrum Pylori in Man, . 292
Robinson, Mr. James, the Adapted Forceps, . . . 439

S.

Salter, S. James A., on Dental Pathology, . . . 14
Sercombe, Mr., Destruction of the entire Palate treated by mechanical means, . 102
Stones, Polishing, . . 154
Salivary Calculus in the Duct of Wharton, . . . 344
Supernumerary Teeth, Osseous Union, . . . . 451
Spence, James, Poisoning by Chloroform, . . . 264
Smith, H. R., on Models and Casts, . . . . 272
Spinal Marrow, the true sympathetic, . . . . 289
School Hygeine, . . . 298
St. Louis Society of Dental Surgeons, . . . . 600
Shepherd, J. M., on Cheoplastic Dentistry, . . . 550

T.

Teeth, Influence of Climate upon the, . . . 74
Tumor, Removal of a, from the Hyoid Bone, . . . 132
Teeth, Remarks on Supernumerary, . . . . 305
Tumors, Essay on, in the Palatine Region, . . 324, 456
Teeth, Minute Anatomy of, 348
Teal, Thomas P., on Plastic Operations, . . . . 442
Teeth, Canine, with two roots, 451
Tobacco and Alcohol, Physiological Effects of, . . 229
Teeth, Hayes' Method of Mounting, . . . . . 269
Taft on Excavators, . . 276
Teeth, Cheoplastic Method of Mounting, . . . 302

Murray-

PAGE

U.

Ure, Dr., Obituary, . . 304

V.

Volck, on Filling Labial Surfaces of Upper Incisors, 322

PAGE.

W.

Watt, on the Action of Topical Remedies on Inflamed Dentine, . . . . . 112
Watt, on Dental Chemistry of the Mouth, . . . . 281
Widom Teeth, Affections Determined by, . . . . 339
Western Dental Society, . 335
Woman's Hospital Association, 599
Warren, J. M., on Tumors of the Parotid Region, . . 587

